# Ebola Response Impact on Public Health Programs, West Africa, 2014–2017

**DOI:** 10.3201/eid2313.170727

**Published:** 2017-12

**Authors:** Barbara J. Marston, E. Kainne Dokubo, Amanda van Steelandt, Lise Martel, Desmond Williams, Sara Hersey, Amara Jambai, Sakoba Keita, Tolbert G. Nyenswah, John T. Redd

**Affiliations:** US Centers for Disease Control and Prevention, Atlanta, Georgia, USA (B.J. Marston, E.K. Dokubo, A. van Steelandt, L. Martel);; US Centers for Disease Control and Prevention, Monrovia, Liberia (D. Williams);; US Centers for Disease Control and Prevention, Freetown, Sierra Leone (S. Hersey);; Ministry of Health and Sanitation, Freetown (A. Jambai);; Ministry of Health, Conakry, Guinea (S. Keita);; Ministry of Health, Monrovia (T.G. Nyenswah);; US Centers for Disease Control and Prevention, Albuquerque, New Mexico, USA (J.T. Redd)

**Keywords:** Capacity building, outbreak, Ebola virus, Ebola, epidemic, response, global health security, hemorrhagic fever, public health, international cooperation, investments, West Africa, Guinea, Liberia, Sierra Leone, viruses, Ebola virus disease, EVD

## Abstract

Events such as the 2014–2015 West Africa epidemic of Ebola virus disease highlight the importance of the capacity to detect and respond to public health threats. We describe capacity-building efforts during and after the Ebola epidemic in Liberia, Sierra Leone, and Guinea and public health progress that was made as a result of the Ebola response in 4 key areas: emergency response, laboratory capacity, surveillance, and workforce development. We further highlight ways in which capacity-building efforts such as those used in West Africa can be accelerated after a public health crisis to improve preparedness for future events.

The Ebola epidemic that was first recognized in 2014 and ravaged the West Africa countries of Liberia, Sierra Leone, and Guinea was a stark illustration of the risks that emerging pathogens and epidemic-prone diseases pose to local and global health security in settings that had limited public health capacity. More than 28,000 Ebola cases were reported from the 3 countries during the epidemic, and >11,000 persons died (*1*). These countries are among the least developed in the world (*2*), and their weak infrastructures and underfunded health systems were further compromised by the epidemic. During the initial months of the Ebola epidemic, limited capacity to rapidly identify suspected cases, confirm diagnoses, and implement preventive measures contributed to widespread transmission (*3*). By the time control was achieved, there had been widespread, devastating impacts on those infected and their families, as well as on the nations’ healthcare systems and economies (*4*) and population health (*5*). Control of the outbreak required substantial effort from host country governments and populations and crucial resources and inputs from multilateral and bilateral partners, nongovernmental organizations (NGOs), and individual persons from outside the 3 countries. In usual circumstances, establishing public health systems and capacities to detect, prevent, and respond to urgent global health threats requires long-term planning and investment (*6*). However, the swift and massive response to this epidemic established methods and resources that are transferable to responses to other health threats, affording an unparalleled opportunity for more rapid expansion of emergency response capacities than would usually be possible in such settings. 

We describe public health progress that was made as a result of the Ebola response in 4 key areas: emergency response, laboratory capacity, surveillance, and workforce development. We then reflect on the challenges and opportunities of supporting this progress immediately after the large public health response.

## Emergency Response

Although response coordination was challenging, especially during the initial phase, establishment of incident management systems (IMS) for the Ebola response facilitated coordination of multiple partners that contributed to control of the main outbreak. In Liberia, the Ministry of Health (MOH) established a national IMS in July 2014, with support from the US Centers for Disease Control and Prevention (CDC), the World Health Organization (WHO), and other partners. Management of daily activities through Emergency Operations Centers (EOCs) improved coordination of response efforts at national and county levels (*7*). During the response, the physical location for the national EOC moved from a temporary location to a new permanent infrastructure on the campus of the MOH. In Sierra Leone, outbreak response was coordinated primarily through national and district Ebola response centers supported by civilian and military personnel and resources from the United Kingdom. During the response, new infrastructure was created to increase coordination capacity, emergency response coordination plans were developed, and designated staff were trained. In Guinea, the IMS was coordinated through a Guinea-led National Coordination Cell with support from WHO, CDC, and the Public Health Agency of Canada. An EOC was established, and staff received basic training in emergency management that facilitated coordination efforts.

The appearance of Ebola clusters after continuous transmission was controlled provided evidence that Ebola virus could persist in survivors of Ebola virus disease (EVD) and could be sexually transmitted to others, initiating new chains of transmission (*8*–*10*). Therefore, it was essential to maintain capacity to rapidly recognize and respond to Ebola cases. The first well-characterized case of transmission related to viral persistence occurred in Liberia, ≈1 month after the epidemic had first been controlled and before Liberia had met the WHO criteria to be declared free of Ebola transmission (*8*,*9*). At that point, the response structure and resources remained in place. The diagnosis was rapidly confirmed, the response was robust, and there was no evidence of secondary transmission. 

Additional clusters (2 in Liberia, 3 in Sierra Leone, and 1 that began in Guinea and spread to Liberia) occurred after interruption of transmission in each country (Figure). The responses to these additional clusters were also robust; in most instances, transmission was limited to 0 or 1 generation (*11*). In Sierra Leone, responses to 2 clusters were coordinated through the same structures used to respond to the main epidemic. The responsibility for emergency response coordination was transferred to the Ministry of Health and Sanitation on January 1, 2016. The agency’s abilities were immediately tested by the recognition of an EVD case, likely related to transmission from an EVD survivor, on January 14, 2016 (*12*). The Ministry of Health and Sanitation stood up its emergency response structure and led a complex control effort that required coordination across 5 districts (*13*). The response led to identification of 131 contacts and implementation of enhanced community surveillance in 1 district for 2 months after the end of contact monitoring. The cluster was limited to 1 generation; disease occurred only in the index case-patient and a single high-risk contact.

**Figure:**
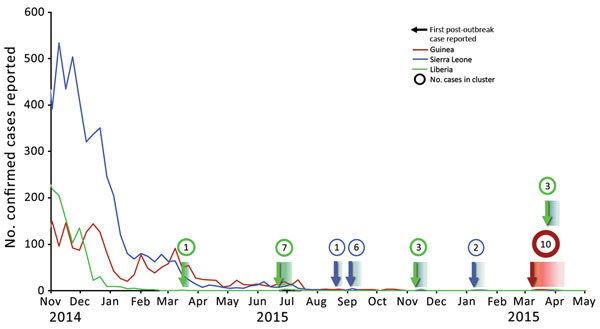
Ebola virus disease clusters after interruption of the 2014–2015 Ebola outbreak in Liberia (green), Sierra Leone (blue), and Guinea (red). Lines reflect total weekly case numbers during the primary outbreak. Arrows indicate the first reported case in each postoutbreak cluster; color indicates the country where the cluster was first recognized (the March 2016 cluster began in Guinea, but spread to Liberia), and gradients indicate timespan of cluster. Circle sizes are proportional to cluster size, and the total number of confirmed and probable cases in each cluster is shown in the circles.

The final cluster of Ebola during the epidemic was recognized in March 2016 (*10*) and occurred under conditions that were similar to the initial situation in the main epidemic; cases were first diagnosed in southeastern Guinea, and a person with a history of high-risk contact fled across the border to Liberia, where Ebola was confirmed in a patient at a hospital in the capital, Monrovia. Responses were led by host country government IMSs and supported by a range of international partners. Although the cluster in Guinea was not identified until there had already been 3 generations of viral transmission, and initial contact identification efforts were delayed by community resistance, the response was effective, containing spread to 2 additional generations. In Liberia, transmission was limited to 1 generation, affecting only immediate family members of the index case-patient. These outcomes were vastly different than that for the initial introduction of Ebola in West Africa.

IMSs have proven beneficial for response efforts beyond those for which they were originally established. The effective control of the Ebola outbreak in Nigeria after travel of an infected person from Monrovia to Lagos in July 2014 was facilitated by the use of an established polio IMS (*14*). Likewise, IMSs established for the Ebola response have provided a structure for organization of other response efforts. In Liberia, increases in the number of measles and Lassa fever cases led to the activation of the IMS on March 14, 2016; the IMS coordinated case investigations, contact tracing, diagnostic evaluation, case management, and prevention efforts. In Sierra Leone, the IMS was activated for an outbreak of measles, which was successfully controlled after a vaccination campaign, and for investigation of cases of acute flaccid paralysis. In Guinea, the EOC established during the Ebola response was integrated into the newly formed Agence National de Sécurité Sanitaire (ANSS) and is responsible for managing epidemics in Guinea; the EOC is managed by a dedicated team of 5 ANSS staff members assisted by CDC, Public Health Agency of Canada, and NGO partners. The EOC has been activated to coordinate investigations and responses to yellow fever and measles outbreaks and provides strong support to the surveillance unit of the ANSS by coordinating meetings and information sharing, producing situational reports, and providing logistic support.

## Expansion of Laboratory Capacity

At the beginning of the Ebola epidemic in West Africa, diagnosis relied on complex tests, primarily reverse transcription PCR (RT-PCR), conducted in carefully controlled settings. Capacity to conduct these tests in Liberia, Sierra Leone, and Guinea was limited, and the initial diagnosis was confirmed by testing of samples sent to an international reference laboratory. During the outbreak, most samples were tested by international teams in field laboratories (*15*). However, over the course of the outbreak, capacity to conduct RT-PCR was established or expanded in national laboratories in each country, and capacity for new technologies was developed, including the use of the GeneXpert platform (Cepheid, Sunnyvale, CA, USA) for PCR and rapid diagnostic tests (RDTs) based on lateral flow assays. These new tests became critical for confirmation of cases later in the outbreak and for ruling out disease and supporting Ebola surveillance. For example, hospital staff performed testing that identified the first case in Liberia during the final outbreak in early 2016 (*16*). In Guinea, Ebola RDTs were used to expand testing capacity to a broader patient population than would have otherwise been tested and to screen for infection status among the deceased to allow families to proceed rapidly with burial. OraQuick Ebola Rapid Antigen Tests (OraSure Technologies, Inc., Bethlehem, PA, USA) were piloted at 15 sites in Forécariah Prefecture in October 2015 by the Guinea MOH and Red Cross staff (*17*) and eventually used to test >4,000 febrile patients and >3,000 deceased patients. Although there appears to be potential for a useful role for Ebola RDTs, progress has been limited for availability, liscensing, and development of guidelines for use of these tests, and the role of postoutbreak Ebola-specific RDTs has diminished. As of late 2017, Liberia, Sierra Leone, and Guinea maintain national and sometimes regional capacity to conduct EVD testing.

In all 3 of these countries, the expansion of Ebola diagnostic capacity extended beyond the ability to diagnose acute infection. Serologic testing contributed to the understanding of disease transmission (*18*), and programs were established that supported testing of semen and other body fluids for Ebola virus RNA (*19*). Local determination of viral sequences also provided key information to inform control efforts; for example, a laboratory established in Sierra Leone in April 2015, staffed by locally trained scientists, conducted rapid sequencing of full Ebola RNA genome sequences and informed the investigation of subsequent Ebola clusters (*13*,*20*).

Expanding Ebola diagnostic capacity improved capacity for diagnosis of other diseases of public health importance, and there has been substantial progress in developing or updating laboratory strategic plans, establishing and improving sample transport networks, and safely storing biologic specimens. In Liberia, diagnostic capacity has been established or reestablished for all identified priority reportable diseases (Table 1), and a nationwide sample transport system has transported >50,000 laboratory specimens from >302 sites across all 15 counties since April 2015. In Sierra Leone, focused collaborative efforts have improved the infrastructure at the national reference laboratory and supported broad training in quality management; progress in establishing systems for sample transport has been limited. In Guinea, much of the laboratory equipment and infrastructure used for Ebola RT-PCR diagnosis by international partners, including a field laboratory established by the US Defense Threat Reduction Agency, have been donated to the MOH. Multiple partners are assisting the MOH to expand diagnostic capacity on these platforms to other diseases of epidemic potential.

**Table 1:** Timeframe for establishment or reestablishment of capacity to test for key notifiable diseases in Liberia after 2014–2015 Ebola outbreak and response*

IDSR priority disease	Q1 2016	Q2 2016	Q3 2016	Q4 2016	Q1 2017
Acute flaccid paralysis	Sent to WHO/regional laboratory outside Liberia for testing				
Acute watery diarrhea (cholera)	†	†	‡	§	§
Acute bloody diarrhea (shigella)	†	†	‡	§	§
Human rabies	Sent to WHO/regional Laboratory outside Liberia for testing				
Lassa fever	†	†	‡	§	§
Measles	‡	§	§	§	§
Meningitis	†	†	†	‡	§
Neonatal tetanus	Not applicable; diagnoses based on clinical symptoms				
Viral hemorrhagic fever (including Ebola virus disease)	§	§	§	§	§
Yellow fever	‡	§	§	§	§

*IDSR, Integrated Disease Surveillance and Response framework; Q, quarter, WHO, World Health Organization. †Capacity was not available during the specified quarter. ‡Capacity was partially established. §Established laboratory capacity.

## Improved Surveillance

Before the Ebola epidemic, sentinel and event-based disease surveillance systems were generally limited in all 3 affected countries; these were further disrupted by the epidemic. However, the Ebola response and health system recovery efforts in these countries have led to improved surveillance for EVD and other epidemic-prone diseases. Event-based surveillance in Liberia is implemented as part of a broad system for Integrated Disease Surveillance and Response (IDSR), which documents 14 priority diseases and conditions. A 5-year IDSR strategic plan is in place and surveillance officers at national and subnational levels have undergone training based on updated IDSR technical guidelines (*21*). Through the Community Event-Based Surveillance system (https://www.globalcommunities.org/liberia), events in the community are reported to a surveillance focal person at the closest health facility, then to the district and county surveillance officers, and reported weekly to the MOH Disease Prevention and Control unit at the national level. Timeliness and completeness of reporting were high before the Ebola outbreak, fell during the outbreak, and currently average >99%. Efforts are ongoing to improve the quality of both the reported data and the response to reports of notifiable diseases and to implement an electronic early warning system to further improve alert notification and response.

Since the Ebola outbreak in Sierra Leone, IDSR-based surveillance has been implemented nationwide. Although IDSR had been technically adopted by Sierra Leone, its implementation had been incomplete before the Ebola outbreak. Improvements in the quality of data collation, analysis, and presentation by central public health authorities have been supported through training, mentorship, and supportive supervision that has included comprehensive data quality audits. The system now monitors 28 priority diseases, conditions, and events. Surveillance data are reported electronically in all 13 districts by using a mobile electronic Integrated Disease Surveillance and Response system (eIDSR) that is compliant with the DHIS2 data management system (https://www.dhis2.org/); this system resulted in 94% of health facilities reporting to their districts in 2016 (Table 2).

**Table 2:** Improvements in the timeliness and completeness of routine district surveillance reporting after 2014–2015 Ebola outbreak and response, Sierra Leone, 2015–2016*

Health district	November 8–4, 2015					May 29–June 4, 2016		
	No. district HFs	No. (%) HFs reported to district		Timeliness		No. district HFs	No. (%) HFs reported to district	Timeliness
Kambia	68	30 (44†)		T§		69	67 (97)	T§
Port Loko	106	0 (0†)		NR†		111	102 (92)	T§
Bombali	104	0 (0†)		NR†		113	111 (98)	T§
Koinadugu	72	24 (33†)		T§		72	63 (88)	T§
Tonkolili	103	0 (0†)		NR†		107	96 (90)	T§
Kono	86	80 (93)		T§		91	91 (100)	T§
Kenema	123	26 (21†)		T§		123	(120 98)	T§
Kailahun	86	16 (18†)		T§		86	85 (99)	T§
Bombali	121	38 (31†)		T§		128	128 (100)	T§
Moyamba	100	95 (95)		T§		101	101 (100)	T§
Bonthe	55	54 (98)		T§		55	50 (91)	T§
Pujehun	77	0 (0†)		NR†		77	47 (61‡)	T§
Western Area	114	65 (57‡)		L‡		120	118 (98)	T§
Overall	1,215	428	35	NC		1,253	1,179 (94)	NC

*Timeliness indicates timing of districts reporting to national level. During 2015, 35% of HFs in Sierra Leone reported Ebola cases to their respective districts. During 2016, 94% of health facilities reported to their districts, and all districts reported at the national level. Data source: CDC Sierra Leone Country Office analysis of Sierra Leone Ministry of Health data. HF, health facility; L, late; NR, no report; NC, not calculated; T, on time. †Level of completeness <50%; performance did not meet minimum standard.  ‡Level of completeness >50% and <80%; performance met minimum standard, but did not meet target. §Level of completeness >80%; performance met target.

In August 2015, Guinea’s MOH created and validated the Surveillance of Epidemic-Prone Diseases plan. In February 2016, IDSR training was conducted for national trainers, who then trained other surveillance system staff. Also in early 2016, Guinea established a novel program to monitor for Ebola resurgence. Ebola survivors were engaged in active surveillance for Ebola-like illness among their contacts and in their communities (Surveillance Active en Ceinture SA-Ceint [*22*]). During the final months of the Ebola epidemic, the MOH also launched community-based surveillance for epidemic-potential diseases in priority prefectures, which supported reporting of key community-level alerts to the local health facility. A DHIS2-based eIDSR reporting system was established for collection of monthly surveillance data in all 38 prefectures of Guinea; the eIDSR system is being expanded in 2017 to include the weekly and immediate surveillance reporting, including case-based surveillance for priority diseases.

## Expansion of Human Capacity

Building public health capacity within the staff of governments is expected to have long-term, broad impacts (*6*). Although it is difficult to precisely measure the effect of a capable public health workforce, quality public health responses are highly dependent on the availability of well-trained staff. When Ebola spread to Nigeria, trained epidemiologists rapidly mounted extensive and successful contact identification and monitoring activities and kept Ebola from spreading broadly, likely preventing a catastrophic outcome (*23*). Thus, a major priority in building public health capacity is to support training in surveillance and epidemic response.

In each of the countries most affected by the Ebola epidemic, the response offered an opportunity to identify persons who have capacity to conduct public health activities. Many persons engaged as surveillance officers during the response demonstrated interest in and aptitude for these activities and have since chosen to pursue training and careers in public health.

Training in field epidemiology is among the top priorities related to expanding public health capacity. Frontline Field Epidemiology Training Programs (FETPs) have been established in all 3 of the countries most affected by the Ebola epidemic. The FETP-Frontline program provides 3 months of on-the-job training and supervision for surveillance officers working within the MOH (*24*). In Liberia, the FETP-Frontline was launched in August 2015; by early 2017, more than 120 surveillance officers in Liberia had completed training, and there are now trained staff in all 15 counties and each of Liberia’s 90 districts. Sierra Leone established a FETP-Frontline program in June 2016 that has now graduated >35 trainees from the national response structure, including all districts. By early 2017, FETP participants had conducted >50 case investigations for acute flaccid paralysis, rabies, maternal deaths, cholera, measles, yellow fever, meningitis, neonatal tetanus, and unexplained deaths, as well as investigations of outbreaks of Lassa fever and rubella. In Guinea, the FETP was launched in December 2016 by the training of 8 MOH staff who will mentor their peers. A cohort of 25 MOH staff began the training program in January 2017; 80 staff are expected to graduate by mid-2018.

FETP-Intermediate, a 9-month program to train supervisory surveillance officers and strengthen their field epidemiology, data analysis, and public health skills (*24*), was launched in Liberia in April 2017 and in Sierra Leone in mid-2017 and will launch in Guinea in early 2018. This training will equip surveillance officers with knowledge and skills to supervise staff and provide leadership during outbreak responses.

Workforce development has included a broad range of other training activities. In Liberia, Sierra Leone, and Guinea, focused training and mentoring on infection prevention and control (IPC) principles and practices was provided at health facilities in the area of an Ebola cluster by using an approach termed ring-IPC (*25*), and thousands of healthcare workers have been trained in IPC principles. Sierra Leone has initiated workforce capacity building in preservice and in-service training programs in laboratory, epidemiology, infection prevention and control, program management, and emergency management. In Guinea, laboratory training has included diagnosis of EVD, meningitis, cholera, and shigellosis, as well as sample transport, biosafety/biosecurity, quality management systems, and molecular biology. A critical element of workforce development has been to support training of managers responsible for public health programs.

## Effects of the Ebola Response on General Public Health Capacity

The resources committed to the Ebola response and post-Ebola recovery have facilitated improvements in the public health systems in West Africa. Beyond resources, there are several other critical requirements for effective expansion of public health capacity. In a 2008 practice note (*26*), the United Nations Development Programme highlighted the essential nature of the “demand side” of the capacity-building equation: the requirement that host countries value and support the need to invest in the identified capabilities. Throughout the epidemic, there were examples of uncertainty within national governments and the affected populations about whether the Ebola threat was real (*27*) but also evidence of growing appreciation of the need for and the ability to successfully implement control measures. The governments of the affected countries have expressed broad appreciation for the support provided by international partners (*28*).

Effective capacity building also requires trust of those offering support (*26*). Partnerships should be established and expanded transparently and must be based on understanding and mutual responsibility. Development of this type of partnership usually takes years. However, the Ebola epidemic juxtaposed external responders with those from the host country under conditions that demanded close and effective working relationships that could not function without mutual trust and respect. Maintaining effective relationships built during the crisis has likely accelerated progress during postepidemic recovery. Successful, locally led responses to new clusters of Ebola and to conditions such as measles and acute flaccid paralysis demonstrate the potential for a crisis such as the Ebola epidemic to lead to improvements in local capacity that can have long-lasting benefits, improving health security for the affected nations and the world.

Since the development of the Joint External Evaluation (JEE) tool (*29*), progress toward compliance with 2005 International Health Regulations (*30*) can now be assessed systematically. Liberia and Sierra Leone were among the 25 countries that completed initial JEEs by the end of 2016 (*29*); a JEE was completed in Guinea in April 2017. Progress was evaluated by comparison with previously conducted self-assessments; all 3 countries achieved acceptable levels of compliance in several areas assessed by the JEE and clear progress in others. The JEE is not meant to be used to compare countries; however, the performance measures in the Ebola-affected countries were consistent with those achieved by several countries that had higher development indexes.

There are serious risks to the progress that has been achieved in the region. All 3 Ebola-affected countries continue to receive crucial ongoing support from international donors and technical partners. However, although the US government maintains a high priority for supporting global health security activities (*31*), critical funding to support critical activities, such as surveillance, laboratory capacity, and workforce development, was provided through a one-time emergency appropriation (*32*). It will likely not be possible for the US government and its partners to maintain the staffing in West Africa that was established in the wake of the outbreak. Neither is it certain that resources for capacity building from other donors will be sustained. Although surveillance systems currently continue to provide timely data on critical disease threats, it may not be possible to maintain community-based activities that were established during or after the Ebola outbreak. The gains made in laboratory capacity are especially fragile; laboratories in all 3 countries continue to rely on support from partners for equipment maintenance and replacement, reagents, and ongoing training. Local laboratory capacities and sample transport function remain suboptimal, and there is persistent need for international partners to provide reference laboratory testing, as was the case for the May 2017 outbreak of meningococcal meningitis in Liberia (*33*).

Clearly, it is ideal to build public health capacity before the occurrence of a public health threat. However, there are lessons from the post-Ebola capacity-building efforts to strengthen global health security. Donors and organizations that support an emergency response should be reassured that resources committed to a response—if appropriately coordinated and targeted—can have an impact beyond the response itself. When possible, continuing support into the postepidemic period could both optimize readiness for possible resurgence of the initial threat and contribute to broad and rapid progress toward health security goals.

## Conclusions

Global health security relies on the ability of all countries to prevent, rapidly detect, and respond to public health threats at their source. The West Africa Ebola epidemic highlighted the importance of strong public health systems and the need for local public health systems that include ongoing surveillance, a well-trained workforce, laboratory capacity, and emergency response capabilities. In settings with limited public health capacity or in which the magnitude of a health threat overwhelms local capacity and requires international support, response efforts provide a unique opportunity for strengthening public health systems and can serve as a further catalyst to accelerate progress toward global health security goals.
